# P297: Increasing needle stick injuries amongst the radiology team-needs attention!

**DOI:** 10.1186/2047-2994-2-S1-P297

**Published:** 2013-06-20

**Authors:** A Ghafur, PR Vidyalakshmi, K Chandra, P Kannaian, J Sahaya

**Affiliations:** 1 Infectious diseases, Apollo speciality hospitals, Chennai, India

## Introduction

Needle-stick injuries and blood contacts pose a risk of pathogen transmission during invasive procedures. As interventional radiological procedures for diagnostics and therapeutics are on rise, reinforcing importance of sharp disposal in this population is mandatory.

## Objectives

Analysis of the vulnerable area for needle stick injuries.

## Methods

Analysis of 24 month data of needle stick injury as documented by the hospital infection control committee from a neurosurgical and oncology tertiary care hospital in Chennai, South India.

## Results

Twenty one needle stick injuries were documented in the last two years. Six of them were doctors and nurses, 4 were radiology technicians and 4 were ward assistants and 1 was a phlebotomist. Five episodes occurred on venepunture 4 were following biopsy and four while clearing clinical waste, 5 during line insertion .Four of the sources were positive for blood borne viruses, two were HIV positive one HCV and one hepatitis B. All the patients received Post exposure prophylaxis at the earliest (2 ART,1 Booster with Hepatitis B vaccine).None of them had seroconversion on follow up. Needle stick injuries occurred most commonly during radiological procedures (33.3%) and in the CCU (33.3%).

## Conclusion

As interventional radiological procedures are increasing , this area along with the CCU needs to be targeted for CME or educational interventions.

## Competing interests

None declared

